# Photophysical and Primary Self-Referencing Thermometric Properties of Europium Hydrogen-Bonded Triazine Frameworks

**DOI:** 10.3390/molecules27196687

**Published:** 2022-10-08

**Authors:** Chaoqing Yang, Dimitrije Mara, Joydeb Goura, Flavia Artizzu, Rik Van Deun

**Affiliations:** 1 Department of Chemistry, Zhejiang University, Hangzhou 310027, China; 2 ZJU-Hangzhou Global Scientific and Technological Innovation Center, Hangzhou 311215, China; 3 L3—Luminescent Lanthanide Lab, Department of Chemistry, Ghent University, Krijgslaan 281-S3, 9000 Ghent, Belgium; 4 Institute of General and Physical Chemistry, Studentski trg 12/V, 11000 Belgrade, Serbia; 5 Engineering Science & Technology Division, CSIR-North-East Institute of Science & Technology, Jorhat 785006, Assam, India; 6 Department of Sustainable Development and Ecological Transition (DISSTE), University of Eastern Piedmont “A. Avogadro”, Piazza S. Eusebio 5, 13100 Vercelli, Italy

**Keywords:** luminescence, lanthanides, hydrogen bonding, HOFs, thermometry

## Abstract

Lanthanide hydrogen-bonded organic frameworks (LnHOFs) are recently emerging as a novel versatile class of multicomponent luminescent materials with promising potential applications in optics and photonics. Trivalent europium (Eu^3+^) incorporated polymeric hydrogen-bonded triazine frameworks (PHTF:Eu) have been successfully obtained via a facile and low-cost thermal pyrolysis route. The PHTF:Eu material shows a porous frame structure principally composed of isocyanuric acid and ammelide as a minor constituent. Intense red luminescence with high colour-purity from Eu^3+^ is obtained by exciting over a broad absorption band peaked at 300 nm either at room or low temperature. The triazine-based host works as excellent optical antenna towards Eu^3+^, yielding ~42% sensitization efficiency (*η_sens_*) and an intrinsic quantum yield of Eu^3+^ emission (*Φ_Eu_*) as high as ~46%. Temperature-dependent emission studies show that PHTF:Eu displays relatively high optical stability at elevated temperatures in comparison to traditional inorganic phosphors. The retrieved activation energy of 89 meV indicates that thermal quenching mechanisms are attributed to the intrinsic energy level structure of the metal-triazine assembly, possibly via a thermally activated back transfer to ligand triplet or CT states. Finally, by using an innovative approach based on excitation spectra, we demonstrate that PHTF:Eu can work as a universal primary self-referencing thermometer based on a single-emitting center with excellent relative sensitivity in the cryogenic temperature range.

## 1. Introduction

Hydrogen-bonded nanomaterials are attractive hosts for a wide range of biological guests, such as biomacromolecules and biocatalysis agents. Recently, the emerging class of hydrogen bonded organic frameworks (HOFs) based on the molecular self-assembly via non-covalent interactions, mainly hydrogen bonding, of organic building blocks, have demonstrated a set of properties that makes them promising for biological fluorescence labelling, sensing, anti-counterfeiting, and illumination. The constitutional units of HOFs are usually highly rigid molecules featuring aromatic moieties with a large π-conjugated system, which are typically suitable to yield interesting luminescent properties [1]. Depending on the degree of π-conjugation, organic compounds can capture and emit photons in tunable wavelength ranges, which facilitates the rational design of luminescent HOF materials with versatile colors. Moreover, the structure of these frameworks widely involves a variety of intermolecular interactions including electrostatic, π-π stacking, and van der Waals forces, enabling charge transfer transitions between the constituting π-conjugated systems which could significantly affect and enhance the emission [1,2,3,4,5]. For example, the hydrogen-bond-mediated supramolecular interactions between different chain moieties in polymer carbon dots allow for electronic transitions between spatially separated molecular orbitals leading to emission enhancement [5].

However, despite the high versatility of luminescent HOFs, their emission properties are characterized by low “color-purity” (broad emission bands), relatively small Stokes’ shifts, and typical nanosecond-ranged lifetimes, limiting their potential biosensing/biolabelling and temperature sensing applications. Trivalent lanthanide cations (Ln^3+^), on the other hand, have very narrow absorption and emission lines, showing large apparent Stokes’ shifts, long emission decay times and excellent response to temperature changes [6]. These features have made lanthanide luminescence of great importance in a variety of applications, encompassing not only hi-tech photonic devices, but also potential biological immunoassays and non-contact temperature probes [6,7,8]. However, Ln^3+^ ions’ luminescence is a result of transitions within the partially filled 4f shells. As these transitions are parity-forbidden, this leads to low molar absorption coefficients resulting in inefficient optical excitation and weak emission [9]. Our previous researches have shown that HOFs can be conveniently used as a tunable platform for constructing multi-emissive lanthanide (Ln) molecular materials [10,11,12,13]. Despite the interest in these materials, there are still few reports about the description of the fundamental photophysical properties of lanthanide ions in doped HOFs and limited understanding has been achieved on the structure/properties relationship of these materials. Moreover, the ratiometric temperature sensing properties of lanthanide-doped HOFs have been very scarcely investigated and mostly based on the dual emission from the matrix and Eu^3+^ in a restricted temperature range [14]. However, the poor intrinsic structural/morphological definition, the high flexibility, and the extreme sensitivity to external stimuli, such as moisture or various chemical species, of such doped polymeric materials, could easily lead to instability of the temperature sensing performances which would require constant accurate calibration.

Here, we report a Eu^3+^-incorporated polymeric hydrogen-bonded triazine framework (PHTF:Eu) synthesized by a facile solid phase thermal pyrolysis route and provide an insight on the factors that govern its emission efficiency via photoluminescence (PL) steady state and time-resolved measurements as well as absolute quantum yields and temperature-dependent PL studies. Moreover, we investigate the potential application of this material as a ratiometric primary and self-referencing thermometer in the 10–410 K temperature range based on a universal approach that relies on the photoluminescence excitation (PLE) spectra instead of the PL-based conventional method. This approach, which solely takes into account Eu^3+^-centered lines, is particularly suitable for this class of doped HOFs materials as it allows for applying single-center-based ratiometric temperature sensing while bypassing the often uncontrolled ligand-to-metal, metal-to-metal, and spurious external energy transfer as well as possible inhomogeneous ligand/crystal field effects, yielding an univocally defined thermometric equation.

## 2. Results and Discussion

### 2.1. Structure and Morphology of PHTF:Eu

**Figure 1:**
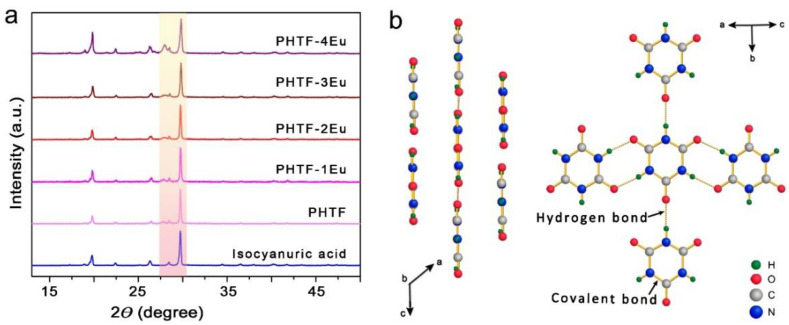
(**a**) Normalized PXRD patterns of PHTF, PHTF:Eu, and pure isocyanuric acid; (**b**) Scheme showing how an isocyanuric acid monomer connects to other neighboring in-plane or out-of-plane units through eight N–H···O H-bonds. Covalent bonds are shown as saffron yellow solid lines and H-bonds as yellow dotted lines.

The PHTF material and its Eu^3+^-doped derivatives PHTF:Eu of different Eu^3+^ concentrations were synthesized at 255 °C via a solid phase thermal pyrocondensation reaction from a precursor obtained from a wet reaction between Eu^3+^ and urea (U) labelled as U-Eu (the detailed description is given in the Experimental Section). In order to identify the structure of the as-synthesized material, PXRD measurements were carried out at room temperature. The PXRD pattern of the pristine PHTF matrix shown in Figure 1a is consistent with the characteristic peaks of isocyanuric acid. Two well-resolved peaks at lower angles, 19.8° and 22.3°, are attributed to the in-planar packing between isocyanuric acid molecules through multiple N−H···O H-bonds [15]. Another strong broad peak at 29.7°, corresponding to a *d*-spacing of 0.32 nm supports the π-π stacking of individual isocyanuric acid nanolayers as shown in Figure 1b [16]. All PHTF:Eu samples exhibit similar reflection peaks as the pristine PHTF. With the increase in Eu^3+^ doping, a diffraction peak at 29.7°, significantly decreases in intensity, which might be due to the Eu^3+^ ions affecting the in-plane packing of H-bonded molecules [10]. The enlarged PXRD pattern shown in Figure S1 reveals that the obtained PHTF contains a minor amount of ammelide. No characteristic peaks of Eu_2_O_3_ and Eu(NO_3_)_3_·xH_2_O were found in PHTF-4Eu.

**Figure 2:**
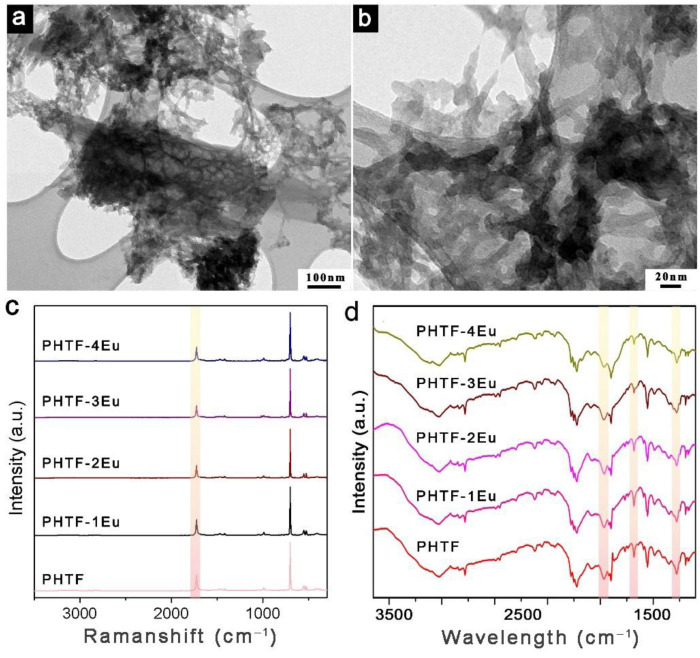
(**a**,**b**) TEM images of PHTF-2Eu at different magnifications; (**c**) Normalized FT-Raman spectra of PHTF and PHTF:Eu; (**d**) Normalized DRIFTS spectra of PHTF and PHTF:Eu.

The CHN elemental analysis, shown in Table S1, confirmed the observations made from the PXRD results. The PHTF has a composition in close proximity with the theoretical wt % of cyanuric acid. The slightly high nitrogen content in PHTF should originate from the ammelide impurity. Upon increasing the Eu^3+^ loading, the carbon, hydrogen, and nitrogen content of the PHTF:Eu gradually decreased, which might be due to the increase in structure defects in the PHTF framework [10]. The N/C ratio slightly increases due to the polycondensation of isocyanuric acid into ammelide at temperatures exceeding 250 °C [17]. The actual Eu^3+^ concentration in PHTF:Eu was investigated by ICP-MS analysis and the results show that the maximum content achieved in PHTF-4Eu is below 9% wt (Table S2). The morphologies of the PHTF and PHTF:Eu samples were investigated by SEM and TEM microscopy. The pristine PHTF as well as the PHTF:Eu samples have a similar but quite irregular morphology consisting of small flakes and large agglomerates with particle sizes between 0.3 and 5 µm (Figures S2 and S3). The TEM images show that PHTF (Figure S2) as well as PHTF-2Eu (Figure 2a,b) present an irregular hollow structure with different cavity volumes. The blocky structure is slightly cracked after Eu^3+^ ions doping. The STEM-EDX mapping confirmed the homogeneous distribution of Eu^3+^ in the PHTF structures (Figure S2e–h).

FT-Raman spectroscopy has been used to investigate the molecular and lattice vibrations of PHTF. As shown in Figure S4a, the spectrum of pristine PHTF is in good agreement with the characteristic peaks of the solid isocyanuric acid which displays a series of peaks located at 525, 552, 703, 991, and 1725 cm^−1^. The most intense peak at 703 cm^−1^ is related to the ring out-of-plane bending vibration. Another peak at 1725 cm^−1^ could be ascribed to the C=O stretching vibration [11]. The normalized FT-Raman spectra of pristine PHTF and Eu^3+^-doped PHTF are given in Figure 2c. The peak at 1725 cm^−1^ originating from the C=O stretching vibration steadily weakens upon increasing the Eu^3+^ concentration, which suggests that the C=O group effectively coordinates europium ions. The enlarged FT-Raman curves shown in Figure S4b reveals that a peak at 1015 cm^−1^ attributed to the ring breathing vibration of ammelide molecules becomes more pronounced, which is in accordance with the XRD results. No evidences of residual urea, Eu(NO_3_)_3_, or Eu_2_O_3_ nanoparticles are found for PHTF:Eu, as shown in Figure S4c. Similar information can be drawn from the DRIFTS spectra shown in Figure 2d. The stretching vibration of the carbonyl group at 1500 cm^−1^ and the ring vibration bands at 770 and 1200 cm^−1^ were found to become progressively weaker for the PHTF:Eu samples [15,18]. These results further hint at a dentate bridging coordination mode for the PHTF units through C=O groups coordinating Eu^3+^ ions [13].

**Figure 3:**
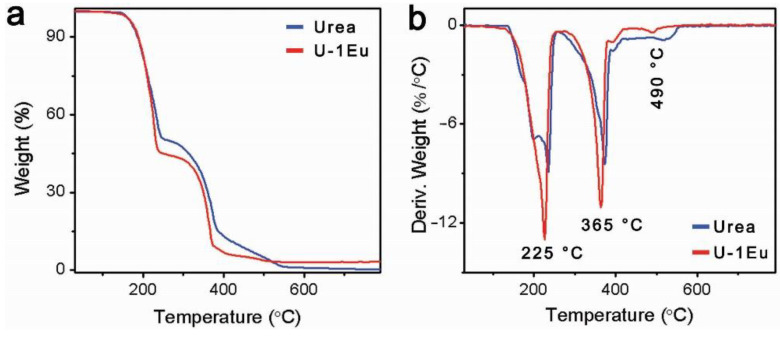
(**a**) TG and (**b**) DTG curves of urea and U-1Eu.

In order to get more insight into the pyrolysis mechanism of PHTF:Eu, thermogravimetric (TG) analysis was carried out under air. It is known that, upon heating, urea undergoes condensation reactions with elimination of ammonia [17]. The TG curve of pure urea (Figure 3a) exhibits mass losses in three stages. The first major mass loss of about 50.3% can be attributed to the rapid deamination of urea forming isocyanuric acid and a small quantity of ammelide. The second stage corresponds to a thermal condensation process where those compounds are transformed into polymeric carbon nitride [17]. A third step begins after 400 °C, where all materials gradually sublimate, and the organic components eventually decompose completely. Accordingly, the derivative thermo-gravimetric analysis (DTG) curve of urea shows three distinct DTG peaks, 225 °C, 376 °C, and 519 °C (Figure 3b). The europium trinitrate prominently influences the pyrolysis of urea. Compared to the pyrolysis of pristine urea, the Eu^3+^ doped urea presents more rapid mass losses, which indicates that the incorporated Eu^3+^ ions accelerate the polycondensation process [19].

### 2.2. Emission Colour Purity of PHTF:Eu

**Figure 4:**
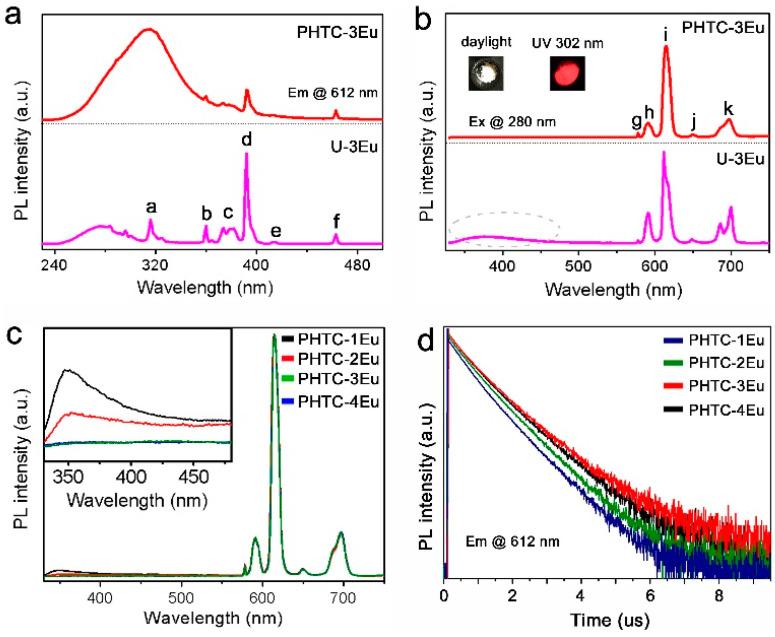
(**a**) Normalized excitation (λ_em_ = 612 nm) and (**b**) emission spectra (λ_ex_ = 280 nm) of PHTF-3Eu and U-3Eu. Insert: photos of PHTF-3Eu under daylight and a 302 nm UV lamp. (**c**) Normalized emission spectra of PHTF:Eu excited at 280 nm, Insert: enlarged emission spectra of PHTF:Eu samples in the range of 330 to 480 nm. (**d**) PL decay curves of PHTF:Eu excited at 280 nm.

An extensive investigation of the photoluminescence (PL) properties of the as-synthesized samples was conducted. The PL properties of Eu^3+^ doped samples were investigated both before (U-3Eu) and after (PHTF-3Eu) heat treatment. In the excitation spectrum of U-3Eu monitored at 612 nm, the most intense emission peak of Eu^3+^, a weak broad band can be seen between 250 and 310 nm, as well as a series of sharp peaks extending from 300 to 500 nm (Figure 4a). These sharp peaks can be reliably attributed to inner 4f shell transitions of Eu^3+^. All labelled peaks have been assigned to appropriate transitions in Table S3. The broad excitation band for U-3Eu can be ascribed to a Eu-O charge-transfer transition (Figure S5a) or ligand-to-metal transitions giving rise to energy transfer from the urea matrix to Eu^3+^ dopants. For PHTF-3Eu, the broad excitation band of higher intensity at longer wavelengths (308 nm) might be attributed to ligand-to-metal transitions (Figure S5b), which indicates a more effective interaction between Eu^3+^ ions and the PHTF matrix [20].

The emission spectra (Figure 4b) of the two samples are observed after exciting into the broad absorption band at 280 nm. It could be clearly seen that both samples yield strong sharp peaks attributed to the characteristic Eu^3+^ f–f transitions. The emission spectrum of U-3Eu presents a broad band in the region of 330–480 nm, which indicates that the energy transfer form urea to Eu^3+^ is not fully efficient. On the other hand, after heat treatment, only the strong f-f transition peaks of Eu^3+^ are observed for PHTF-3Eu. As shown in Figure 4b, PHTF-3Eu shows bright red luminescence with CIE (CIE, 1931) coordinates of (0.657, 0.337) approaching the Rec. 2020 (0.708, 0.292) [21] Red Primary light specification. The normalized emission spectra for PHTF:Eu with different Eu^3+^ concentrations show similar profiles (Figure 4c). However, the matrix emission bands in the 330–480 nm region change and constantly weaken upon increasing Eu^3+^ concentration as a result of the increased number of acceptors. The calculated CIE chromaticity coordinates of PHTF:Eu (Table S4) also clearly show that the concentration of Eu^3+^ ions influences the colour-purity (Figure S5c). The decay dynamics (Figure 4d) of Eu^3+^ in all samples follows a monoexponential trend with retrieved time constants of 523.6, 534.9, 549.2, and 540.9 µs for PHTF-1Eu, PHTF-2Eu, PHTF-3Eu, and PHTF-4Eu, respectively (Table S5). The slight decrease in the decay time constant for PHTF-4Eu might be attributed to a concentration quenching effect of Eu^3+^ ions.

The following discussion on the Eu^3+^ sensitization mechanism and the thermal response of the emission will be focused on PHTF-3Eu as this sample showed the highest emission intensity.

### 2.3. Eu^3+^ Sensitization in PHTF:Eu

The sensitization efficiency (*η_sens_*) from the donor PHTF matrix to Eu^3+^ could be calculated and analysed according to the following Equation (1) [22]:

(1) $$\eta_{\mathrm{sens}}=\frac{\Phi }{\Phi_{\mathrm{Eu}}}$$

where *Φ* is the overall quantum yield which can be readily measured through the use of an integration sphere, and the intrinsic quantum yield of Eu ions (*Φ*_Eu_) which can be deduced by Equations (S1) and (S2). These equations however require the determination of the electric and magnetic transition dipole strengths which are quite difficult to retrieve. However, since the characteristic Eu^3+^ transition, ^5^D_0_ → ^7^F_1_ is only magnetic dipolar in nature, its intensity does not depend on the crystal field. Fortunately, this offers a way to greatly reduce the experimental parameters needed and *Φ*_Eu_ could be calculated from the corrected emission spectrum and time-resolved photoluminescence data via a simplified Einstein’s equation for spontaneous emission (Equation (2)) [22]:

(2) $$\Phi_{\mathrm{Eu}}=\frac{\tau_{obs}}{\tau_{rad}}=\frac{\tau_{obs}\;A_{MD}\;n^{3}\;I_{TOT}}{I_{MD}}$$

where *τ_obs_* is the actual lifetime of the emitting excited state, and *τ_rad_* is the radiative lifetime of this state in absence of any non-radiative de-activation processes. *A_MD_* is the spontaneous radiative rate for the Eu^3+ 5^D_0_ → ^7^F_1_ magnetic dipole transition and is taken as 14.65 s^−1^ [22]. *n* is the refractive index of the medium whose value was taken as 1.748 for cyanuric acid. *I_TOT_*/*I_MD_* is the ratio of the total integrated area of the Eu^3+^ emission spectrum to the area of the ^5^D_0_ → ^7^F_1_ band. Table 1 presents the main photophysical parameters for the investigated samples. As it can be seen, the change in the crystal/ligand field upon thermal treatment and transformation of the organic matrix has a relevant influence on the oscillator strength of Eu^3+^ emission leading to a dramatic decrease in the radiative lifetime.

The absolute overall quantum yield for PHTF-3Eu, directly measured with an integrating sphere, was found to be 19.2% (Figure S6), much higher than in U-3Eu (5.5%). These values are in agreement with the retrieved lifetimes of Eu^3+^ emission at 612 nm, which were found to be 399.2 µs (Table S6) for U-3Eu (Figure S7) and 549.2 µs for PHTF-3Eu. A reliable interpretation for this result is that Eu^3+^ emission in the hydrated urea matrix is strongly influenced by the presence of quenching sites, such as –OH and –NH groups [23,24]. However, the thermal treatment at 265 °C leads to a series of pyrolysis reactions of urea to form isocyanuric acid, yielding a dehydrated PHTF:Eu material. As a result, the PTHF-3Eu sample displays an improved Eu^3+^ intrinsic quantum yield (45.9%) with respect to its urea-based precursor (20.6%). Importantly, a high sensitization efficiency of 41.8% is found for the PHTF-Eu system. This value, although not comparable to the best results obtained in small molecular complexes where all the antenna units are directly coordinated to the lanthanide acceptor [25,26], is significantly higher than in most Eu-doped systems, such as Eu-doped silica nanoparticles (38%) and Eu-doped YPO_4_ nanocrystals capped with an organic antenna (26%) [27,28]. The reason for this enhanced ligand-to-metal energy transfer efficiency mainly relies on two factors. The first is of spectral origin and is related to the high-lying excited ligand donor energy levels, (above 28,000 cm^−1^) associated to a very broad emission (Figure S5b) which ensure an excellent spectral overlap with the Eu^3+^ acceptor absorption through the upper energy levels manifold (^5^D_3_ to ^5^D_0_ at ~24,400–17,200 cm^−1^) (Figure S8a). The second factor is instead related to the overall kinetics of the process. As previously observed in analogous Eu-doped HOFs semiconducting materials [10], the excited state depopulation of the organic moiety can follow different pathways: (i) intramolecular radiative or nonradiative deactivation; (ii) energy transfer to directly coordinated Eu^3+^ ions; and (iii) charge transfer or exciton migration over the network. This latter phenomenon is significantly enhanced by the establishment of H-bonds within the PHTF matrix (Figure 1b) which increases the rigidity of the network and facilitates interring interactions, including π-stacking. The enhanced exciton migration throughout the H-bonded network increases the chances of energy transfer to Eu^3+^ centers working as “localized traps” in doped materials where the donor-acceptor ratio is significantly above unity (>10 in our case), hence leading to improved sensitization efficiency. In addition, the increased rigidity of the system favors intersystem crossing from singlet-to-triplet excited states and suppresses the non-radiative decay channels [23] thus favoring ligand-to-metal energy transfer, which is believed to occur through the excited ligand triplets (Figure S8a).

### 2.4. Temperature-Dependent Emission of PHTF:Eu

**Figure 5:**
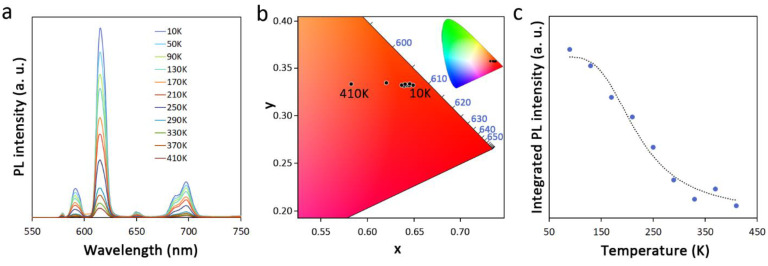
(**a**) Temperature-dependent PL spectra of PHTF-3Eu (λ_ex_ = 300 nm); (**b**) Magnification of a portion of the CIE diagram of PHTF:Eu in the 10–410 K temperature range. The whole diagram is reported in the inset; (**c**) Plot of the integrated PL intensity vs. temperature for the emission band centered at 612 nm (I_02_, ^5^D_0_ → ^7^F_2_) of PHTF-3Eu. The blue circles represent the experimental data, and the dotted curve represents the best fit according to Equation (4) (R^2^ = 0.975).

The thermal response of the PL properties in Ln doped materials is one of the most important parameters for potential applications, and is also fundamental to disclose the structure of energy levels in the material. As shown in Figure 5a, the PL intensity of PHTF-3Eu, taken as a representative example, monotonically decreases with increasing temperature in the range from 10 K to 410 K. No peak shift of Eu^3+^ emission or changes in the full widths at half-maximum (FWHM) are observed. The emissive band related to the PHTF matrix also shows an absolute decrease in intensity, but it gradually enhances with respect to the normalized Eu^3+^ emission (Figure S9) upon increasing the temperature. However, as evidenced in Figure 4b and discussed in the above paragraph, the weak absolute intensity of PHTF-related emission, originated from the efficient sensitization of Eu^3+^, allows PHTF-3Eu to keep a stable emission color purity (Figure 5c) over a broad range of temperatures with relevant deviations of the CIE chromaticity coordinates (Table S7) only at 410 K. The observed thermal stability of the emission color is a beneficial feature for applications in electrically driven optical devices such as LEDs. On the other hand, this behavior does not consent the use of the PL response for a reliable ratiometric temperature sensing based on dual emission [14]. The thermometric properties of PHTF:Eu will be discussed further; nonetheless, important considerations about the thermal quenching mechanism of Eu^3+^ emission can be made here.

The experimental integrated intensity *I* of Eu^3+^ emission from the ^5^D_0_ excited state as a function of the absolute temperature is reported in Figure 5b and Figure S9a. As the temperature increases, some nonradiative channels become thermally activated and this results in a decrease in the luminescence intensity. We fitted the data with a classical Mott-Seitz model [29,30], according to the following Equation (3):

(3) $$I\;\left(T\right)=\frac{I_{0}}{1+A\;exp\;\left(-\frac{E_{a}}{k_{B}T}\;\right)\;}$$

where *I*_0_ is the peak intensity at temperature *T* = 0 K, *A* is a parameter related to the radiative lifetime (*τ_rad_*) as A =*τ_rad_*/*τ*_0_, *E_a_* is the activation energy in the thermal quenching process, and *k_B_* is the Boltzmann constant. The best fitting results were retrieved for the 90 – 410 K temperature range and the resulting curve is reported as a dotted line in Figure 5b. The inclusion of low temperature (10–50 K) data brings significant deviations and lowers the quality of the fit, whereas a consistent improvement is found when fitting with an extended double-term Mott-Seitz model (Equation (S4)) [6], which takes into account the competition of multiple excited-state deactivating channels (Figure S10 and Table S8). However, this model yields results of difficult interpretation and one activation energy value (10 meV) that is physically unreasonable; therefore, in the following discussion, we rely on the fitting results of the classical Mott-Seitz model in the restricted temperature range, as reported in Figure 5c. The retrieved activation energy of PHTF-3Eu of 89 meV (719 cm^−1^) is significantly higher than what was found in undoped polymeric g-C_3_N_4_ materials [31] as well as in many other luminescent semiconducting nanostructures such as ZnO, CdTe, and carbon nanoparticles [32,33,34], indicating a relatively high stability of the luminescence properties on temperature changes (Table 2). In regard to the origin of the thermal quenching, we first note that the relatively low *E_a_* value found is not compatible with a deactivation pathway through multiphonon relaxation related to high energy vibrational modes of OH groups (~3400 cm^−1^) [24]. This observation is also in agreement with the relatively strong dependence of the emission intensity on the temperature, even below 200 K, where high-energy vibrational quanta of organic groups are hardly populated. Likewise, quenching by material defects does not seem plausible in this case given the monoexponential decay dynamics of Eu^3+^ emission from the ^5^D_0_ level (Figure 4d), which typically indicates matrix homogeneity. This leaves the source of thermal quenching to the internal energy level structure of the organic moiety-Eu^3+^ metal ion assembly. Nonetheless, the activation energy found for PHTF-3Eu is much lower than that retrieved for Eu(thd)_3_ (thd = 2,2,6,6-tetramethyl-3,5-heptanedionato) complex in the gas phase (510.8 meV), where the shortening of the Eu^3+ 5^D_0_ lifetime on temperature increase was attributed to the deactivation through a low-lying ligand-to-lanthanide charge transfer (CT) state [35,36]. Our results are, however, fully in agreement with those recently reported for a Eu^3+^ ketoprofen adduct, whose PL data were best fitted with the extended Mott-Seitz model yielding *E_a_*_1_ = 61 meV (494 cm^−1^) and *E_a_*_2_ = 0.5 meV (3.8 cm^−1^) [37]. Despite the triplet excited state level for the triazine moiety lying well above 22,000 cm^−1^ [38], the wide triplet band broadening typically observed in organic antenna sensitized lanthanide compounds [39] can justify thermally activated energy back transfer to the triplet state or to a low-lying CT state involving the organic triazine units (Figure S8) [36], or possibly a competition between these two deactivation channels. A more in-depth interpretation of the temperature-dependent emission quenching will require more extended dedicated studies that will be the subject of future work.

### 2.5. Ratiometric Primary Self-Referencing Thermometric Properties

**Figure 6:**
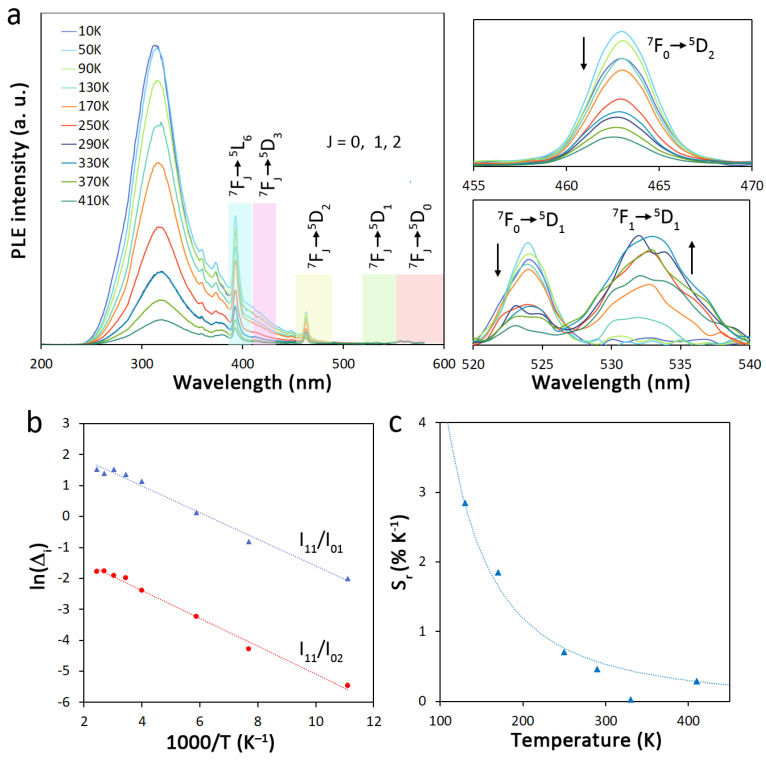
(**a**) Temperature-dependent PLE spectra of PHTF-3Eu monitored at 612 nm. The two insets on the right show a magnification of the bands considered for the assessment of the thermometric properties. The spectra associated to the ^7^F_0_ → ^5^D_2_ transition have been corrected for the overlapping contribution of the broad ligand-centered band; (**b**) Dependence of the natural logarithm of the thermometric parameter lnΔ*_i_* from the inverse of the absolute temperature T. The dotted lines represent the best linear regression fit based on Equation (5) (R^2^ = 0.987 and 0.991 for the blue and red line, respectively); (**c**) Experimental S_r_ % values at different temperatures (blue triangles). The dotted curve represents the best fit to data according to Equation (7) (retrieved Δ*E* = 331 cm^−1^, R^2^ = 0.949).

To assess the potential application of PHTF-3Eu as ratiometric primary self-referencing thermometer, we adopted a recently proposed approach based on the analysis of the photoluminescence excitation (PLE) spectra of Eu^3+^ [40]. As shown in Figure 6a, the broad ligand and the narrow Eu^3+^ PLE bands monitored at 612 nm, corresponding to the most intense Eu^3+^ ^5^D_0_ → ^7^F_2_ emission line, display a strong dependence from temperature. For metal-centered excitation, this is related to the close J energy levels structure of the ground level manifold ^7^F_J_, with *J* = 0, 1…6), where the first three levels, lying within ~1000 cm^−1^ energy separation, are thermally coupled even below 400 K. As the temperature increases, the ^7^F_0_ level population is depleted in favor of the population of the ^7^F_1,2_ levels following a Boltzmann distribution. Under the legit assumption that the integrated intensities of the related excitation bands are proportional to the number of emitters of a certain ^5^D_J_ state fed by a specific ^7^F_J_ level (*J* = 0, 1, 2), a known thermometric equation can be written:

(4) $$\Delta_{i}=A_{i}e^{-\Delta E_{i}/k_{B}T}=\frac{I_{J^{\prime }}}{I_{J}}$$

where the thermometric parameter Δ*_i_* refers to the ratio of the integrated intensities (*I_J′,J_*) of the *i*-th transitions starting from the ^7^F*_J_*_′_ and ^7^F*_J_* (*J*′ > *J*) levels of the low energy manifold, and A*_i_* is a pre-exponential factor depending on the absorption transition rates, the level degeneracy, and the refractive index of the medium. Δ*E_i_* is the energy difference between the ^7^F*_J_*_′_ and ^7^F*_J_* (*J*′ > *J*) levels, which can be easily retrieved from the barycenter of the excitation bands or by experimental data fitting according to Equation (4). The major advantage of using PLE instead of PL is that the temperature dependence of the optical features can be described by a known equation (Equation (4)) with easily retrievable constants, allowing for primary thermometers with internal calibration (self-referencing) of universal application. In fact, the single determination of the thermometric parameter Δ*_i_*_0_ at a T_0_ temperature univocally defines Equation (4). In addition, PLE spectra are usually not affected by crystal field splitting, hence overcoming band broadening due to a distribution of emitters in different environments (as in doped inorganic, hybrid, and polymeric materials) and require low energy excitation sources thus preventing sample heating (affecting the system response to temperature changes). Therefore, the proposed approach is fully suitable to be applied for the material under investigation. To study the ratiometric thermometric properties of PHTF-3Eu, we took primarily into consideration the bands associated to the ^7^F_0_ → ^5^D_1_ (525 nm) and ^7^F_1_ → ^5^D_1_ (535 nm) transitions since they do not overlap with other bands and are intense enough to be quantifiable. As additional reference, we also considered the ^7^F_0_ → ^5^D_2_ associated band (463 nm), since its relatively high intensity allows the subtraction of the contribution of the overlapping tail of the broad ligand-centered band without relevant error. As expected (Figure 6a), the intensity of the bands related to the ^7^F_0_ starting level decreases whereas that related to the ^7^F_1_ level increases with some deviations at temperatures above 370 K likely due to the increasingly sizeable thermal coupling of the ^7^F_2_ and ^7^F_3_ levels.

Two thermometric parameters were calculated as the ratio between the integrated intensities of the band related to the ^7^F_1_→^5^D_1_ transition (I_11_) to the integrated intensity of the ^7^F_0_ → ^5^D_1_ (I_01_) and ^7^F_0_ → ^5^D_2_ (I_02_) transitions and the retrieved experimental data were then fitted with Equation (4) in the linear form:

(5) $$ln\Delta_{i}=lnA_{i}-\frac{\Delta E_{i}}{k_{B}}\frac{1}{T}$$

As shown in Figure 6b, curve fitting returns two nearly parallel lines with ΔE_01_ = 300 cm^−1^ and 313 cm^−1^ for Δ_01_ = I_11_/I_01_ and I_11_/I_02_, respectively, which are very close to the value retrieved from the PLE spectrum (322 cm^−1^) and to the value calculated for the free ion (379 cm^−1^) [25]. To further assess the potential of PHTF-3Eu as primary thermometer with self-referencing attributes, we also calculated Δ_00_ (=I_02_/I_01_) taking into account transitions from the same initial level, where Δ*E_i_* = 0 and Equation (4) reduces to Δ*_i_* = *A_i_*. Results shown in Figure S11 demonstrate the independence of this thermometric parameter from temperature, which univocally defines *A_i_* and represents an extremely useful internal calibration to discriminate spurious effects from different matrixes/environments of the thermometer.

Another relevant parameter in luminescence thermometry is the relative sensitivity *S_r_*_,__*i*_, which expresses the variation of the thermometric parameter Δ*_i_* with temperature, and is usually defined as:

(6) $$S_{r,i}=\frac{1}{\Delta_{i}}\left|\frac{d\Delta_{i}}{dT}\right|,$$

which, after differentiation, can be written as:

(7) $$S_{r,i}=\frac{\Delta E_{i}}{k_{B}}\frac{1}{T^{2}},$$

The experimental *S_r_* values for the thermometric parameter calculated from the I_11_/I_01_ ratio on dependence of the temperature are reported in Figure 6b, whereas Figure S12 reports data for the I_11_/I_02_ thermometric parameter. Differently from PL-based thermometers, PLE-based ones yield a monotonic decrease in *S_r_* instead of a usually observed maximum, which results from the absence of any (temperature-dependent) offset in the thermometric Equation (4) (Δ = *Ae*^−Δ*E*/*kT*^ + *B*) [41]. Therefore, the experimental *S_r_* values could be well fitted with the known Equation (7) (dotted lines in Figure 6b and Figure S10) which is demonstrated to yield reliable general predictions of *S_r_* at different temperatures independently from the measurement conditions (matrix/environment). This is also further confirmed by the linear trend of experimental *S_r_* values with the inverse temperature squared 1/*T*^2^ reported in Figure S13. Based on all these considerations, we can conclude that PHTF-3Eu can be successfully employed as a ratiometric self-referencing primary thermometer, particularly in the cryogenic temperature range (100–200K).

## 3. Materials and Methods

### 3.1. Materials and Measurements

All chemicals were purchased from Sigma-Aldrich (St. Louis, MO, USA), Fisher Scientific (Waltham, MA, USA), or Sinopharm (Beijing, China) and used as received without further purification. Powder X-ray diffraction patterns were measured on a Thermo Scientific ARL X’TRA or Bruker D8 diffractometer at room temperature. Diffuse reflectance infrared Fourier transform spectroscopy (DRIFTS) measurements were recorded on a Thermo Nicolet 6700 spectrometer, equipped with a nitrogen-cooled MCTA detector and a KBr beam splitter at room temperature. Scanning electron microscopy micrographs were obtained on a SEM apparatus, type S-3000N (Hitachi). Transmission electron microscopy (TEM) was carried out by using a Cs-corrected JEOL JEM2200FS transmission electron microscope operated at 200 kV. Thermo-gravimetric analysis (TGA) and the derivative thermogravimetry (DTG) was carried out on a Netsch STA 449E3 Jupiter analyzer. Fourier transform Raman spectroscopy (FT-Raman) measurements were recorded on a Thermo Nicolet 6700 NXR FT-Raman spectrometer with an InGaAs detector at room temperature. The CHN elemental analyses were carried out using a thermo organic elemental analysis flash 2000 apparatus. The samples’ photographs were acquired using a Canon IXUS digital camera or iPhone 6s Plus mobile. The samples were placed under a Cole-Parmer laboratory UV lamp (254 or 302 or 365 nm) or irradiated by a Xe900 lamp. For the analysis of the Ln^3+^ concentrations, inductively coupled plasma mass spectrometry (ICP-MS) was carried out with a Nexion 350 spectrometer (Perkin Elmer, Akron, OH, USA). Steady state and transient photoluminescence (PL) at room temperature were performed by using an Edinburgh FLSP920 spectrophotometer equipped with a Hamamatsu R928P PMT detector. The steady state PL spectra were acquired with a 450W xenon lamp. The transient PL experiments were performed using a microsecond flashlamp (repetition rate 0.1–100 Hz) or a pulsed nanosecond EPLED laser (wavelength 331 nm). The temperature dependent measurements were performed using an ARS CS202-DMX-1SS closed cycle cryostat at low temperature range. Temperature dependent steady-state PL and PLE data were analyzed (peak integration, baseline subtraction, curve fitting) using Origin Pro 8.5 software. Spectral data were reported with reference to wavenumbers (cm^−1^) prior to any analysis.

### 3.2. Synthesis of PHTF and PHTF:Eu Hydrogen Bonded Heptazine Framework

A total of 3.03 g (50 mmol) of urea was dissolved in 50 mL of deionized water under stirring at 95 °C. 1.67 mL (0.1 mol/L) of Eu(NO_3_)_3_ solution was added to the hot urea solution. Subsequently, the mixtures were magnetically stirred at 90 °C. Then, the products were dried at 95 °C for 12 h. The sample was crushed in a mortar to get a fine white precursor and labeled as U-1Eu. Then, U-1Eu was heated at a rate of 10 K/min within a muffle furnace to 255 °C under air, and kept for 90 min. The samples were cooled down passively within the furnace. In order to remove the possible residuals of unreacted urea, the obtained raw sample was further washed with deionized water and centrifuged several times. The product was additionally annealed at the same temperature (255 °C) for another 20 min under air. After the sample had been cooled down passively within the furnace, it was crushed. The final product was labelled as PHTF-1Eu. Samples with different molar ratios of urea/Eu^3+^ in 1:0, 200:1, 100:1 and 50:1 ratios were also synthetized. The samples were labelled as PHTF, PHTF-2Eu, PHTF-3Eu, and PHTF-4Eu, respectively.

## 4. Conclusions

In summary, trivalent europium ions incorporated polymeric hydrogen-bonded triazine composites have been developed. These materials can be synthesized via a facile and low-cost solid phase thermal pyrolysis route. Under excitation with UV light, the materials emit bright red luminescence of high colour purity. The PL quantum yield for PHTF-3Eu is 19.2%, much higher than the value obtained for the urea-based precursor U-3Eu (5.5%), due to the high sensitization efficiency (41.8%) and intrinsic quantum yield (45.9%) in the dehydrated system. The PHTF:Eu samples can keep stable colour purity in the temperature range from 10 K to 410 K. The activation energy of PHTF-3Eu, retrieved through temperature-dependent PL measurements, is found to be ~89 meV, which is a value much higher than many popular inorganic and organic emitters, indicating a high stability of the emission intensity upon temperature increase. A thermal deactivation mechanism through crossover via energy back transfer to the ligand triplet state or to a low-lying ligand-to-metal CT state is proposed to account for the emission intensity dependence on temperature. The luminescence thermometric properties of PHTF:Eu have been investigated by adopting a novel approach based on the use of excitation spectra, which yielded a fully univocally determined thermometric equation of general validity. It has been demonstrated that the presented Eu^3+^ doped hydrogen bonded triazine frameworks can be successfully employed as ratiometric primary self-referencing thermometers with excellent sensitivity in the cryogenic temperature range.

## Figures and Tables

**Table 1:** Main photophysical parameters of U-3Eu and PHTF-3Eu.

Parameter	U-3Eu	PHTF-3Eu
*τ_RAD_*	1.943 ms	1.196 ms
*τ_OBS_*	0.399 ms	0.549 ms
*I_TOT_*/*I_MD_*	6.6	10.7
*Φ*	5.5%	19.2%
*Φ*_Eu_	20.6%	45.9%
*η*_sens_	26.7%	41.8%

**Table 2:** Activation energies of various emitting materials.

Material	*E_a_* (meV)	Reference
CdTe	47	[33]
ZnO	59	[34]
carbon nanoparticles	42.7	[32]
Polymeric carbon nitride	73.6	[31]
PHTF:Eu	89	This work
Eu(thd)_3_ (in gas phase)	510.8	[36]

## Data Availability

The data presented in this study are available on request from the corresponding author.

## Supplementary Materials

The following supporting information can be downloaded at: https://www.mdpi.com/article/10.3390/molecules27196687/s1, Table S1: CHN analysis, Figure S1. PXRD patterns, Table S2: ICP-MS analysis, Figure S2: SEM images and STEM-EDX elemental mapping, Figure S3: DLS data, Figure S4: FT-Raman spectra, Figure S5: Additional room temperature PL data, Table S3: Assignment of Eu^3+^ peaks, Table S4: CIE chromaticity coordinates at room temperature, Table S5: Fitting results of the decay curves, Photophysical Parameters discussion, Figure S6: Quantum yield determination spectra, Figure S7: PL decay curves of U-3Eu, Table S6: fitting results of the decay curve of U-3Eu, Figure S8: Energy levels diagrams, Figure S9. Normalized emission spectra at different temperatures, Figure S10. Additional data and fittings for the thermal dependence of the integrated PL intensity, Table S7: Fitting parameters for the thermometric equation, Table S8: CIE coordinates at different temperatures, Figure S11: Dependence of Δ_02/01_ from the temperature, Figure S12: S_r_% for Δ_01_ = I_11_/I_02_, Figure S13: Linear trend of experimental Sr% values with 1/T^2^.

## Sample Availability

Samples of the compounds PHTF and PHTF:Eu cited in this work are available from the authors upon reasonable request.
