# Supplementary material for: Photophysical and Primary Self-Referencing Thermometric Properties of Europium Hydrogen-Bonded Triazine Frameworks
Source: Molecules. 2022 Oct 8;27(19):6687. doi: 10.3390/molecules27196687 (PMC9572016; doi:10.3390/molecules27196687)
Supplement: Supplementary file 1 [file molecules-27-06687-s001.zip › molecules-1933805-supplementary.pdf]

Supporting Information

Photophysical and Primary Self-Referencing Thermometric Properties of Europium Hydrogen-Bonded Triazine Frameworks

Chaoqing Yang, Dimitrije Mara, Joydeb Goura, Flavia Artizzu* and Rik Van Deun

Table S1: CHN analysis of investigated samples, and referenced cyanuric acid, ammelide.

Sample	N (wt %)	C (wt %)	H (wt %)	N/C
cyanuric acid	32.54	27.89	2.32	1.17
PHTF	34.96	27.79	2.66	1.26
PHTF-1Eu	34.01	27.28	2.55	1.25
PHTF-2Eu	30.19	23.64	2.23	1.28
PHTF-3Eu	28.69	21.86	2.20	1.31
PHTF-4Eu	26.75	19.21	2.11	1.39
ammelide	43.72	28.11	3.12	1.56

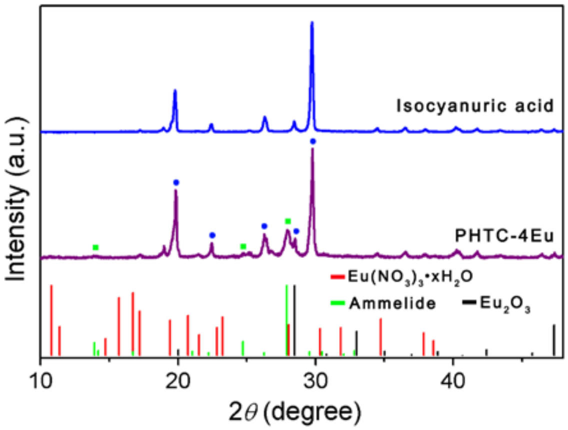

Figure S1. PXRD patterns of PHTF-4Eu and pristine isocyanuric acid. [Vertical bars at the bottom are the position of ammelide (JCPDS 31-1527), Eu_2O_3 (JCPDS 34-0392), and $Eu(NO_3)_3 \cdot xH_2O$ (JCPDS 42-0693)].

Table S2: Actual Eu^{3+} contents for the investigated samples determined by ICP-MS analysis.

Sample	Eu content (wt %)
PHTF-1Eu	2.19 %
PHTF-2Eu	5.12 %
PHTF-3Eu	6.41 %
PHTF-4Eu	8.73 %

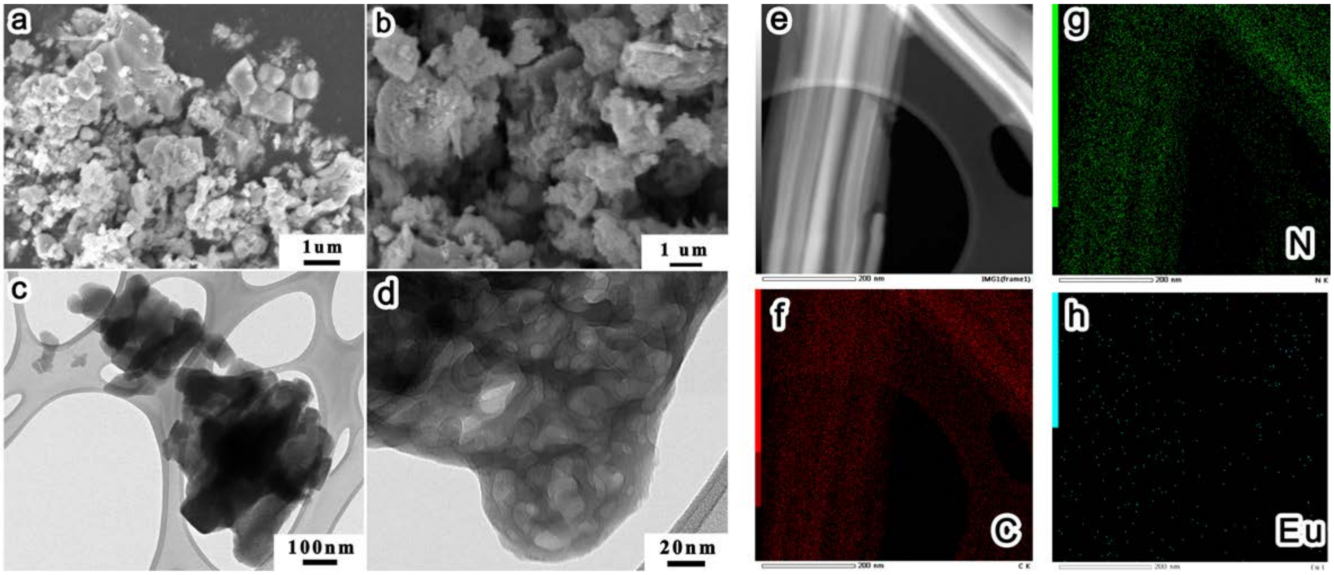

Figure S2. SEM images of (**a**) PHTF and (**b**) PHTF:Eu. (**c**) and (**d**) TEM images of PHTF at different magnifications. (**e-h**) STEM-EDX elemental mapping of PHTF:Eu.

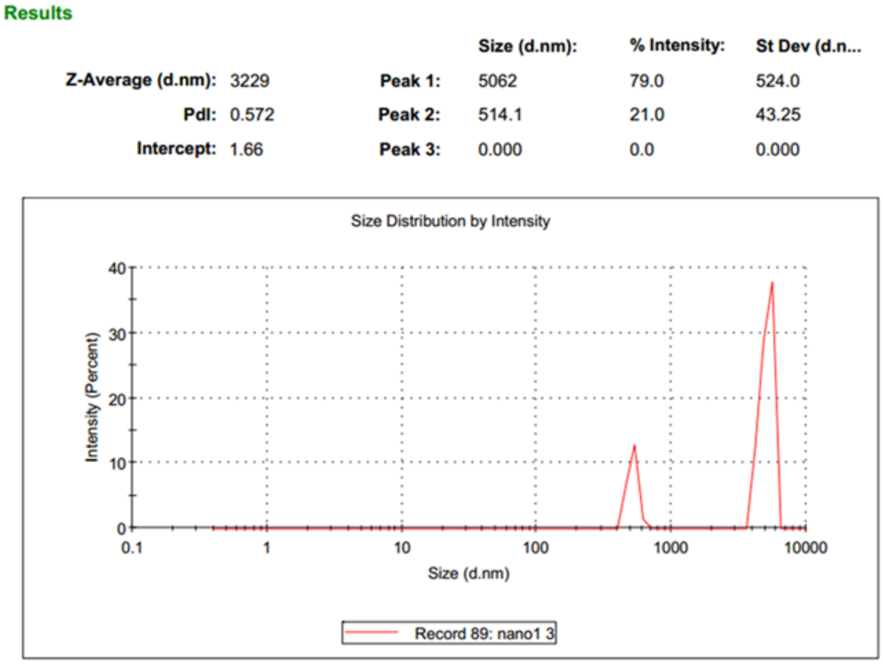

Figure S3. Dynamic light scattering (DLS) results of PHTF:Eu.

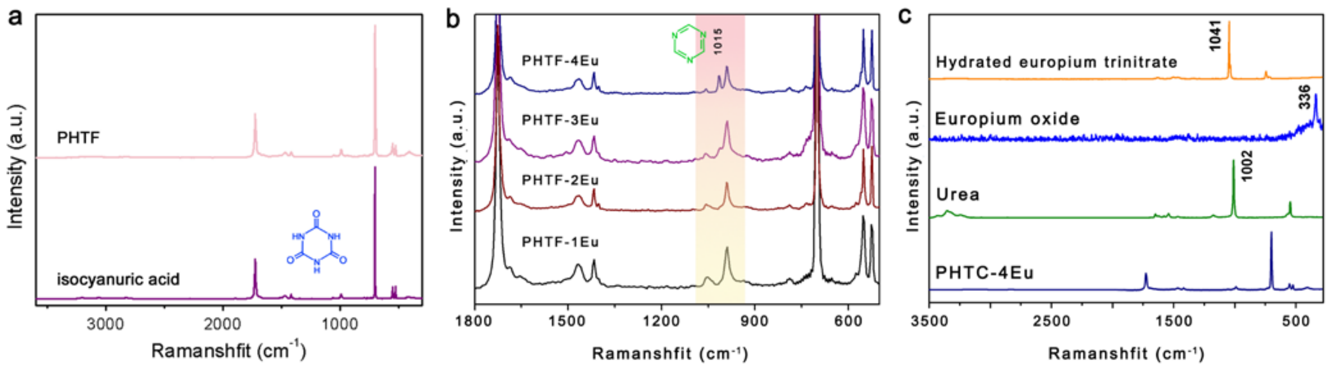

Figure S4. FT-Raman spectra of (**a**) isocyanuric acid and PHTF, (**b**) PHTF:Eu, and (**c**) PHTF-4Eu, urea, Eu_2O_3, and $Eu(NO_3)_3 \cdot xH_2O$.

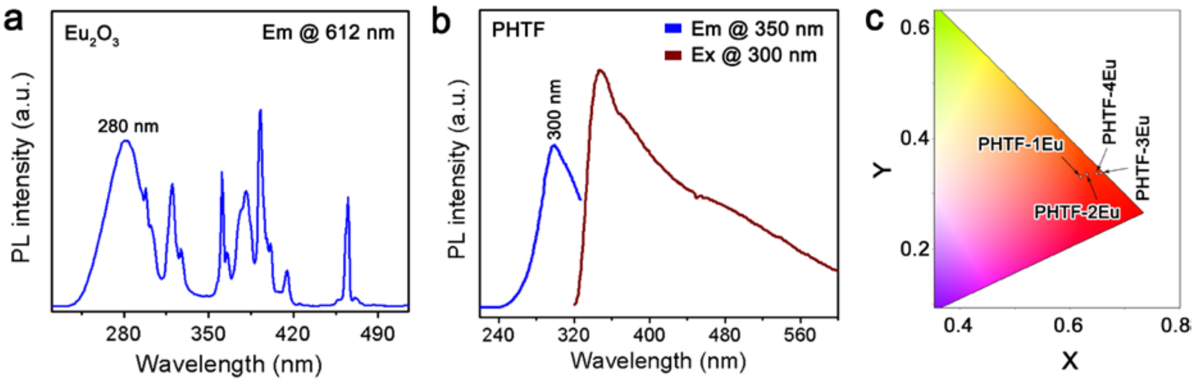

Figure S5. (**a**) Excitation spectrum) of pure Eu_2O_3 (λ_{em} = 612 nm. (**b**) Normalized emission spectrum (λ_{ex} = 300 nm) and excitation spectrum of PHTF (λ_{em} = 350 nm). (**c**) CIE chromaticity diagram of PHTF:Eu excited at 280 nm.

Table S3: Assignment of labelled peaks in the excitation and emission spectra of investigated samples.[1]

Label	Wavelength (nm)	Wavenumber (cm^{-1})	Transition
		Excitation	
a	316	31646	$^5H_6 \leftarrow {}^7F_0$
b	359	27855	$^5D_4 \leftarrow {}^7F_0$
c	372	26882	$^5L_7, {}^5G_5 \leftarrow {}^7F_1$ / $^5G_{2,4,6} \leftarrow {}^7F_0$
d	393	25445	$^5L_6 \leftarrow {}^7F_0$
e	414	24155	$^5D_3 \leftarrow {}^7F_1$
f	462	21645	$^5D_2 \leftarrow {}^7F_0$
		Emission	
g	578	17301	$^5D_0 \rightarrow {}^7F_0$
h	591	16921	$^5D_0 \rightarrow {}^7F_1$
i	614	16287	$^5D_0 \rightarrow {}^7F_2$
j	652	15337	$^5D_0 \rightarrow {}^7F_3$
k	695	14389	$^5D_0 \rightarrow {}^7F_4$

Table S4: CIE chromaticity coordinates for investigated samples at room temperature.

Sample	CIE coordinates (x, y)
PHTF-1Eu	(0.620, 0.330)
PHTF-2Eu	(0.631, 0.335)
PHTF-3Eu	(0.657, 0.337)
PHTF-4Eu	(0.649, 0.335)

Table S5 Fitting results of the decay curves of PHTF:Eu excited at 280 nm and monitored at 612 nm.

Sample	τ (μs)	R^2
PHTF-1Eu	523.6	0.997
PHTF-2Eu	534.9	0.996
PHTF-3Eu	549.2	0.995
PHTF-4Eu	540.9	0.995

Photophysical parameters

The intrinsic quantum efficiency of Ln^{3+} ions (Φ_{Ln}) can be calculated with the use of the natural radiative (A_{rad}) and nonradiative (A_{nrad}) rate constants according to the following Equation S1. This calculation requires evaluating the rate constant of a transition between an initial state and a final state. Taking into account only the dipole magnetic and electric transitions, this rate can be expressed as Equation S2, where υ is the energy of the transition in cm^{-1}, h is the Planck's constant, n is the refractive index of the medium, (2J+1) is the degeneracy of the initial state J, D_{MD} and D_{ED} are the magnetic and electric dipole strengths (in $esu^2\ cm^2$), respectively.[2]

$$\Phi_{Ln} = \frac{A_{rad}}{A_{rad}+A_{nrad}} \qquad \text{Equation S1}$$

$$A\left(\Psi_J,\ \Psi'_{J'}\right) = A_{rad} = \frac{1}{\tau_{rad}} = \frac{64\pi^4\upsilon^3}{3h\,(2J+1)}\left[n^3 D_{MD} + \frac{n(n^2+2)^2}{9} D_{ED}\right] \qquad \text{Equation S2}$$

The internal quantum efficiencies were measured using an integrating sphere on the FLSP920 spectrophotometer, and white $BaSO_4$ powder was used as a reference to measure the scattered excitation light. The internal (η_i) quantum efficiencies (QEs) were calculated through the following equation:

$$\eta_i = \frac{\varepsilon}{\alpha} = \frac{\int L_S}{\int E_R - \int E_S} \qquad \text{Equation S3}$$

where ε is the number of photons emitted by the sample and α is the number of photons absorbed by the sample. L_S is the luminescence emission spectrum of the sample; E_R is the spectrum of the scattered excitation light with the $BaSO_4$ reference sample in the sphere; E_S is the spectrum of the scattered excitation light with the actual sample in the sphere. The quantum efficiencies of all samples were measured using the same method and setup.

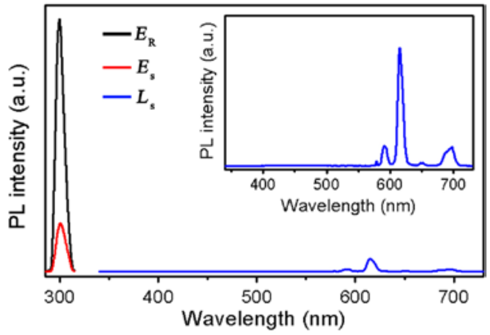

Figure S6. Quantitative excitation and emission spectra of PHTF-3Eu and reference sample measured using an integrating sphere. The inset shows a magnification of the emission spectrum of PHTF-3Eu.

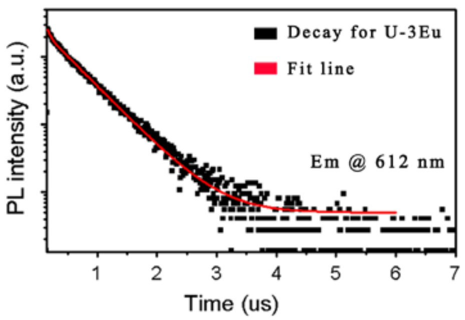

Figure S7. PL decay curves of U-3Eu excited at 280 nm and monitored at 612 nm.

Table S6. Fitting results of the decay curve of U-3Eu excited at 280 nm and monitored at 612 nm.

Samples	τ_1 (μs)	A_1 (%)	τ_2 (μs)	A_2 (%)	τ_{ave} (μs)	R^2
U-3Eu	51.3	70.1	485.4	29.9.	399.2	0.998

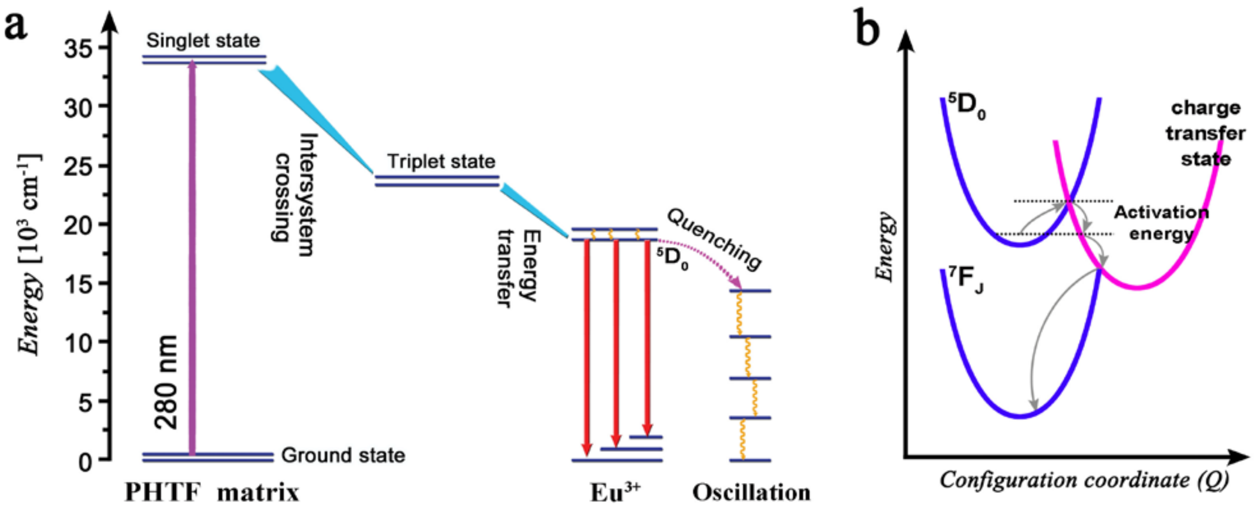

Figure S8. (**a**) Jablonski diagram showing the emission photocycle in PHTF:Eu and the possible quenching mechanisms through multiphonon relaxation. (**b**) Proposed energy decay pathway for the $^5D_0$ state through a low-lying ligand-to-metal charge-transfer state.

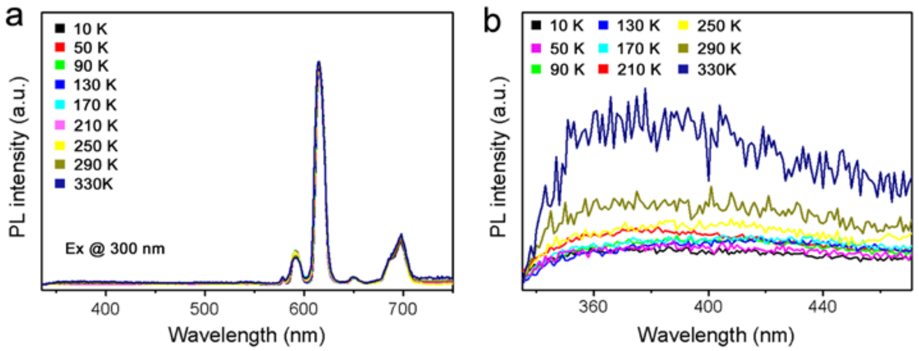

Figure S9. (**a**) Normalized emission spectrum of PHTF-3Eu at different temperatures (λ_{ex} = 300 nm). (**b**) Enlarged spectra in the range from 335 nm to 471 nm.

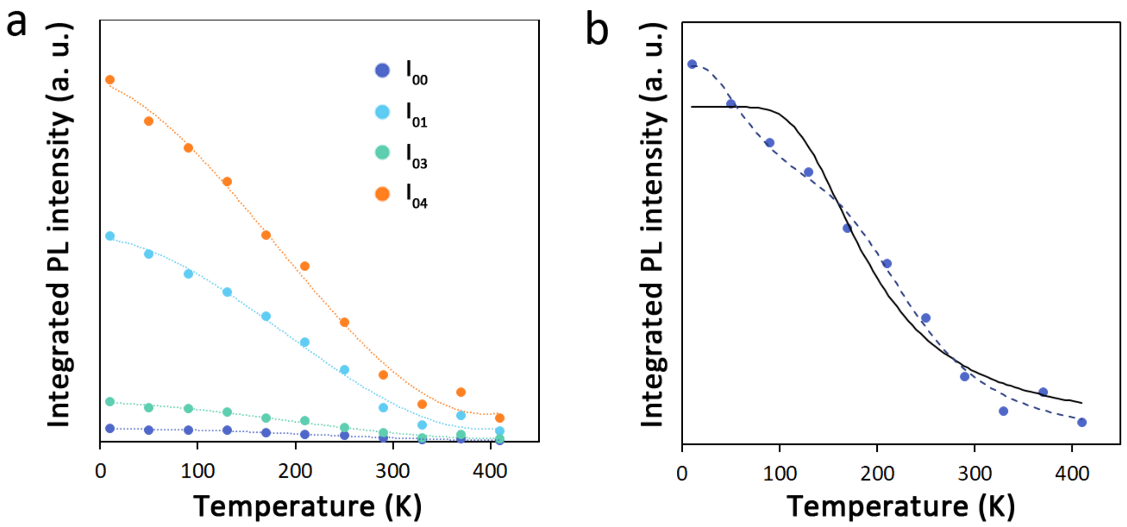

Figure S10. (**a**) Thermal dependence of the integrated PL intensity I_{0J} for the Eu^{3+} $^5D_0 \rightarrow {}^7F_J$ (J = 0, 1, 3, 4) transition emission lines. Dotted lines are intended as a guide. (**b**) Curve fitting of I_{02} experimental values in the range 10 – 410 K according to the Mott-Seitz (Equation 3, solid black line) and extended Mott-Seitz model equations (Equation S7, dotted black line). Fitting parameters are reported in Table S8.

The extended Mott-Seitz equation, which takes into account all the *i*-th nonradiative decay channels, can be written as[3]:

$$I\,(T) = \frac{I_0}{1 + \sum_i A_i \, exp\left(-\frac{E_{a,i}}{k_B T}\right)} \qquad \text{Equation S4 (Extended Mott-Seitz)}$$

Table S7: CIE chromaticity coordinates of PHTF-3Eu at different temperatures (λ_{ex} = 300 nm).

Temperature	CIE coordinates (x, y)	Temperature	CIE coordinates (x, y)
10 K	(0.6491, 0.3323)	250 K	(0.6453, 0.3337)
50 K	(0.6449, 0.3321)	290 K	(0.6202, 0.3348)
90 K	(0.6414, 0.3319)	330 K	(0.6209, 0.3347)
130 K	(0.6393, 0.3316)	370 K	(0.6203, 0.3348)
170 K	(0.6368, 0.3325)	410 K	(0.5825, 0.3335)
210 K	(0.6404, 0.3334)		

Table S8: Fitting parameters for integrated intensity data.

Model	Temperature range	Ea	A	R^2
Mott-Seitz	90-410 K	719 cm-1 (89 meV)	101	0.975
Mott-Seitz	10-410 K	525 cm^{-1} (65 meV)	45	0.955
Extended Mott-Seitz	10-410 K	84 cm^{-1} (10 meV), 946 cm^{-1} (117 meV)	1, 367	0.990

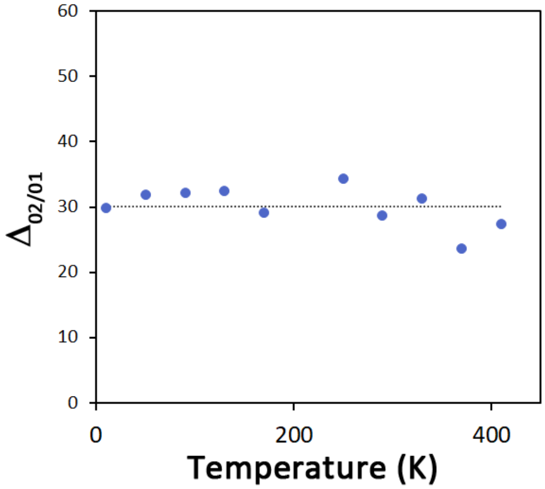

Figure S11. Dependence of the thermometric parameter $\Delta_{02/01}$ from the temperature, where ΔE = 0 (same starting levels). The standard deviation from the mean (dotted black line), taking into account the whole set of values, is 7.7%.

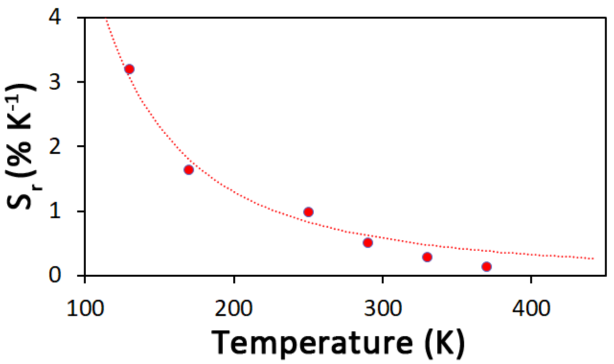

Figure S12. Experimental S_r% retrieved for $\Delta_{01} = I_{11}/I_{02}$ (circles). The dotted curve represents the best fit to data, with retrieved $\Delta E = 361$ cm^{-1} and $R^2 = 0.975$.

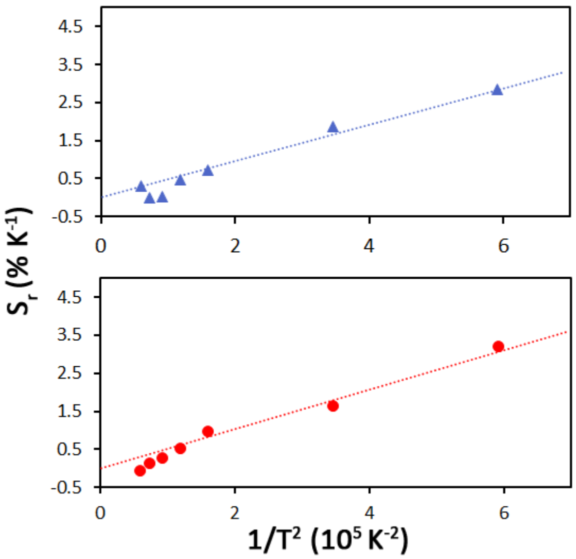

Figure S13. Linear trend of experimental S_r% values for $\Delta_{01} = I_{11}/I_{01}$ (blue triangles, above) and for $\Delta_{01} = I_{11}/I_{02}$ (red circles, below) with $1/T^2$. Dotted lines represents the best linear regression fit to data according to Equation 7 with forced intercept 0.

Supplementary References

1. Carnall, W.; Fields, P.; Rajnak, K. *J. Chem. Phys.* **1968**, *49*, 4424.
2. Aebischer, A.; Gumy, F.; Bünzli, J.-C. G. *Phys. Chem. Chem. Phys.* **2009**, *11*, 1346.
3. Karczewski, G.; Maćkowski, S.; Kutrowski, M.; Wojtowicz, T.; Kossut, J. Photoluminescence study of CdTe/ZnTe self-assembled quantum dots *App. Phys. Lett.* **1999**, *74*, 3011
